# Hypo-Expression of Flice-Inhibitory Protein and Activation of the Caspase-8 Apoptotic Pathways in the Death-Inducing Signaling Complex Due to Ischemia Induced by the Compression of the Asphyxiogenic Tool on the Skin in Hanging Cases

**DOI:** 10.3390/diagnostics10110938

**Published:** 2020-11-12

**Authors:** Aniello Maiese, Alessandra De Matteis, Giorgio Bolino, Emanuela Turillazzi, Paola Frati, Vittorio Fineschi

**Affiliations:** 1 Department of Surgical Pathology, Medical, Molecular and Critical Area, Institute of Legal Medicine, University of Pisa, 56126 Pisa PI, Italy; aniello.maiese@unipi.it (A.M.); emanuela.turillazzi@unipi.it (E.T.); 2 IRCCS (Istituto di Ricerca e Cura a Carattere Scientifico) Neuromed Mediterranean Neurological Institute, Via Atinense 18, 86077 Pozzilli IS, Italy; paola.frati@uniroma1.it; 3 Department of Anatomical, Histological, Forensic and Orthopaedic Sciences, Sapienza University of Rome, Viale Regina Elena 336, 00161 Rome RM, Italy; alessandra.dematteis@uniroma1.it (A.D.M.); giorgio.bolino@uniroma1.it (G.B.)

**Keywords:** immunohistochemistry, c-FLIP, vitality, suicide, hanging

## Abstract

The FLICE-inhibitory protein (c-FLIPL) (55 kDa) is expressed in numerous tissues and most abundantly in the kidney, skeletal muscles and heart. The c-FLIPL has a region of homology with caspase-8 at the carboxy-terminal end which allows the molecule to assume a tertiary structure similar to that of caspases-8 and -10. Consequently, c-FLIPL acts as a negative inhibitor of caspase-8, preventing the processing and subsequent release of the pro-apoptotic molecule active form. The c-FLIP plays as an inhibitor of apoptosis induced by a variety of agents, such as tumor necrosis factor (TNF), T cell receptor (TCR), TNF-related apoptosis inducing ligand (TRAIL), Fas and death receptor (DR). Increased expression of c-FLIP has been found in many human malignancies and shown to be involved in resistance to CD95/Fas and TRAIL receptor-induced apoptosis. We wanted to verify an investigative protocol using FLIP to make a differential diagnosis between skin sulcus with vitality or non-vital skin sulcus in hanged subjects and those undergoing simulated hanging (suspension of the victim after murder). The study group consisted of 21 cases who died from suicidal hanging. The control group consisted of traumatic or natural deaths, while a third group consisted of simulated hanging cases. The reactions to the Anti-FLIP Antibody (Abcam clone-8421) was scored for each section with a semi-quantitative method by means of microscopic observation carried out with confocal microscopy and three-dimensional reconstruction. The results obtained allow us to state that the skin reaction to the FLIP is extremely clear and precise, allowing a diagnosis of unequivocal vitality and a very objective differentiation with the post-mortal skin sulcus.

## 1. Introduction

In humans, defective regulation of the cell death program due to excessive or insufficient apoptosis results in the development of various diseases, including cancer, neurodegenerative diseases and autoimmune diseases [1]. When an organ is injured, a complex, highly-orchestrated healing process starts, requiring the interplay and crosstalk of a multitude of cells and mediators. Each has a histological and biomolecular imprint that have been widely investigated; these are collectively termed as ‘vitality’ and relate to whether or not the victim was alive at the time the trauma was sustained.

A more general mechanism whose importance has only been discovered in recent years is involved in the production of proteins that inhibit the transduction of the apoptotic signal. To prevent uncontrolled cell death or tissue damage, apoptosis is strictly regulated by numerous inhibitors. Many of these inhibitors have been identified because they are expressed by viruses as functional homologues with the task of preventing the death of the host cell. The v-FLICE-inhibitory protein (v-FLIP) produced by the herpes virus inhibits the recruitment and activation of caspase-8 in the death-inducing signaling complex (DISC) [2,3].

The c-FLIPL (55 kDa) is expressed in numerous tissues and most abundantly in the kidney, skeletal muscles and heart. c-FLIPL and c-FLIPS compete with procaspase-8 when highly expressed. Several homologs of FLIP have been found in humans and all appear to have an anti-apoptotic role. Specifically, the cellular FLICE inhibitory protein (c-FLIP) is abundantly expressed in a variety of tumors as an endogenous inhibitor of receptor-induced apoptosis via the caspase-8 pathway [4,5].

The c-FLIP gene resides on chromosome 2q33-34 in a 200-kb cluster that includes both caspase-8 and -10, indicating that these genes are evolved via duplication [6]. The c-FLIP is expressed in three main forms: the long form (c-FLIPL), which is highly homologous to procaspase-8 and -10 and has tandem amino-terminal death-effector domains (DEDs) and a catalytic protease domain inactive at the carboxy-terminal; and the short forms (c-FLIPS and c-FLIPR), which are composed only of the amino-terminal tandem DEDs followed by a short carboxy-terminal tract, with a similar structure to v-FLIP [7,8]. The c-FLIPL has a region of homology with caspase-8 at the carboxy-terminal end which allows the molecule to assume a tertiary structure similar to that of caspases-8 and -10 [9,10].

The Death Effector Domain (DED) present in the c-FLIPL protein binds to the Fas-associated protein with the death domain (FADD) adapter protein, which then recruits c-FLIPL instead of caspase-8 to the receptor complex. Consequently, c-FLIPL acts as a negative inhibitor of caspase-8, preventing the processing and subsequent release of the proapoptotic molecule active form. Therefore, c-FLIPL forms a heterodimer with caspase-8 by binding to it via two domains: DED and caspase-like-domain [11]. Thus, without FLIP, caspase-8 is initially recruited and then activated by an autocatalytic cleavage, with a subsequent cleavage between the large and small subunits and between the caspase domain and the DED of the neighboring caspase. Consequently, the caspase-8 protease active dimer is released into the cytoplasm and initiates the apoptotic cascade [12,13].

At the same time, we acknowledge that gross and histological examination of these marks may sometimes be unreliable and may mislead the forensic pathologist into deciding whether they are due to hanging or post-mortem suspension of the body. For this purpose we wanted to investigate whether the ischemia induced by the compression of the asphyxiogenic tool on the skin is likely to determine the development of hypoxia with consequent hypo-expression of c-FLIP and activation of the caspase-8 apoptotic pathways in the Death-Inducing Signaling Complex (DISC) [14,15].

This mechanism, if validated, could resolve one of the great uncertainties that still reign in the field of forensic pathology, namely the demonstration (not to mention certainty) of the viability of the lesion. In the present study, whether FLIP can be reliably used in forensic practice for differential diagnosis between suicidal hanging and simulated hanging (hanging the victim after murder) was investigated.

## 2. Materials and Methods

### 2.1. Samples

We selected 8 women and 13 men, mean age 52.2 years, who died from suicidal hanging. Regarding the type of hanging material used for ligature, we selected 11 cases in which broad, soft and yielding materials were used and 10 cases in which hard materials used. The knot was situated over the back of the neck (occipital position) in 7 cases, in 4 cases it was situated on the left side of the neck (left mastoid) and in 10 cases on the right side of the neck (right mastoid). Complete hanging occurred in 15 cases and incomplete handing in 6 cases.

We chose as a control group adults (*n* = 13; six women, seven men, mean age 47.3 years) that died from opioid overdose (*n* = 2), car accident (*n* = 3), sudden cardiac death (*n* = 5) and 3 cases of post-mortem suspension of bodies (drug overdose as cause of death in all three cases). These deaths were characterized by their rapidity. The post-mortem interval was ≤36 h in each case. The study was carried out on skin samples. In all cases of hanging, skin sections were removed at the neck in long strips perpendicular to the base of the ligature marks at the site of the major depth of the marks. In control cases skin samples were taken from the anterior face of the neck [16,17].

Only bodies free of post-mortem changes were selected and, according to Italian Law 582/1994 regarding method of assessment and death certification, EKG was performed for 20 min so as to certificate death as soon as possible; therefore, all skin samples were collected within 12–24 h after death.

A routine microscopic histopathological study was performed using haematoxylin-eosin (H&E) staining. In addition, immunohistochemical investigation of skin samples was performed utilizing antibodies anti-FLIP (ab8421), anti-tryptase, anti-CD15, anti-Troponin I fast skeletal muscle.

### 2.2. Immunohistochemical Examination

Our method was as previously published and this study represents an implementation of our previous studies [16,17]. Samples, 8 cm^2^, from each case were fixed in 10% buffered formalin, then washed with phosphate-buffered saline (PBS) and subsequent dehydration was carried out using a graded alcohol series. After dehydration, samples were cleared in xylene, and embedded in paraffin. Sections were cut at 4 μm, mounted on slides and covered with 3-amminopropyltriethoxysilane (Fluka, Buchs, Switzerland).

To test the Anti-FLIP antibody (ab8421), we used resistant prostate cancer and kidney samples. Antigen retrieval was carried out using EDTA buffer in a pressure steamer at 100 °C for 90 min. Slides were stained on an automated immunostainer (Dako Cytomation, Glostrup, Denmark), using a polyclonal anti- FLIP antibody (Abcam clone ab8421 Cambridge, United Kingdom; 1:50 dilution). (Tryptase: 5 min Proteolytic Enzyme (Dako, Copenhagen, Denmark), 20 °C 120 min, 20 °C 1:1000. CD 15: (DAKO, Copenhagen, Denmark) boiling in 0.25 mM EDTA buffer; 120 min, 20 °C 1:50.) Anti-Troponin I fast skeletal muscle (Abcam clone-134,838) was diluted 1:100. After removal of the primary antibodies with three 5-min washes in PBS, sections were incubated for 40 min with biotinylated horse anti-mouse IgG (Vector) diluted 1:200 in 1% NHS. After three 5-min washes in PBS, sections were incubated for 30 min with horseradish peroxidase avidin D (HRP, Vector) diluted 1:1000 with PBS. After three 5-min washes with PBS, the sections were developed with DAB kit (Vector), stopped with rinses of double-distilled water. Bound antibodies were detected with the Dako EnvisionTM System Copenhagen, Denmark. As a negative control, primary antibody was omitted and replaced with PBS. In addition, non- specific rabbit antibody was used, resulting in clean negative results in all cases (not shown). The sections were counterstained with Mayer’s haematoxylin, dehydrated, cover-slipped and observed in a Leica DM4000B optical microscope (Leica, Cambridge, UK) connected to a computerized system with photo camera (DC 480 Leica) [18,19].

### 2.3. Quantitative Analysis

For quantitative analysis, in each immunohistochemical section we made 20 observations in different fields/slides at 100-fold magnification. The samples were also examined under a confocal microscope and a three-dimensional reconstruction was performed (True Confocal Scanner, Leica TCS SPE, Cambridge, UK).

The staining intensity was evaluated using a semi-quantitative immunohistochemical (IHC) scoring scale. A semi-quantitative evaluation of the immunohistochemical findings by two different investigators (MN, AM) without prior knowledge was performed; all measurements were done at the same magnification of image (×10) and the following gradation of the immunohistochemical reaction was used with a scale 0–3, as follows. The amount and extent of marker depletion was scored for each section from 0 to −3:

- 0 = no loss of staining;
−1 = minimal decrease in staining, compared to normally stained tissue;
−2 = clear decrease in staining with some positivity (brown colour) remaining;
−3 = no positive (brown) staining.

The grade was based on the maximum depletion of FLIP noted in the region. The evaluations were carried out separately for each sample, using a double-blind method. In cases of divergent scoring, a third observer decided the final score [16,17].

### 2.4. Statistical Analysis

Semi-quantitative evaluation of the immunohistochemical findings and gradation of the immunohistochemical reaction were described with an ordinal scale and the median value reported. Analysis of variance for the non-parametric data was performed using Kruskal-Wallis test. When differences were found to be significant, analysis between the unmatched groups was elucidated with a Dunn’s Multiple Comparison post hoc test. Significance level was set to 5% (SPSS ver. 16.01 for Windows—SPSS Inc., Chicago, IL, USA).

## 3. Results

Our results allowed us to highlight some interesting and significant data.

The microscopic observation of the skin specimens from hanging marks presented flattening of the epidermal layers, formation of intra-epidermal liquid-filled vesicles, and in a few cases dermal mild leukocyte reactions and alteration of the musculature in the form of Zenker’s necrosis.

In the cases of post-mortem suspension of bodies, plethora of the dermal vessels and metachromasia of the dermal and sub-dermal connective tissue were observed.

**Figure 1:**
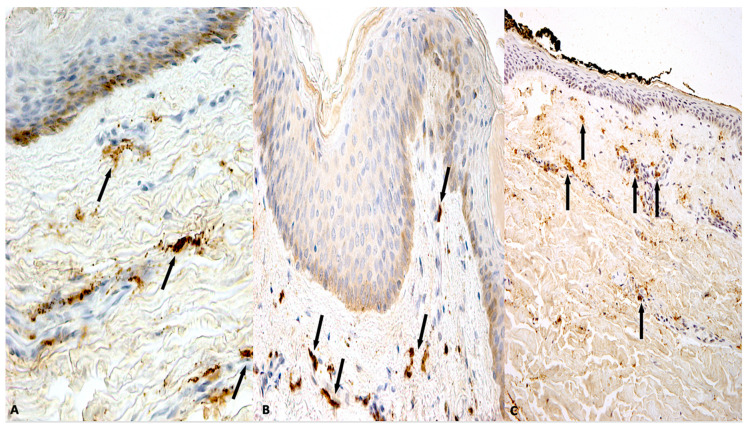
(**A**) Mast cells tagged by tryptase reaction (black arrows) (×60). (**B**) Mast cells appeared numerously, especially near the epidermis, along the blood vessels and in the peri-glandular stroma denoting intense tryptase positivity (black arrows) (×80). (**C**) CD15 reaction (black arrows) to demonstrate a small number of neutrophils near the vessels (early reaction) (×40).

**Figure 2:**
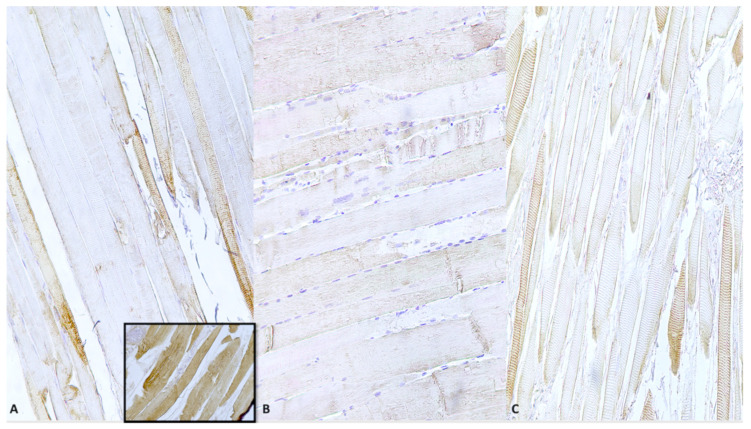
(**A**) Clear decrease in staining with some positivity (brown color) remaining (Grade II) (×100). Insert: positive control. (**B**) Scarce positive TnI fast (brown) intracytoplasmic staining (Grade II) (×100). (**C**) High intracytoplasmic depletion of Troponin I with scarce positivity (Grade II) (×80).

Preliminarily, we confirmed the positivity of the vital sulci with methods that are already established to be reliable markers of vitality, such as the study of tryptase, CD15 and Troponin I fast skeletal muscle, all used as reliable diagnostic tools in forensic practice. In the 21 cases in which the anti-tryptase, anti-CD15 (Figure 1A–C) and anti-troponin I fast skeletal muscle antibodies were used as positive control, we found significant positive reactions (Figure 2A–C).

**Figure 3:**
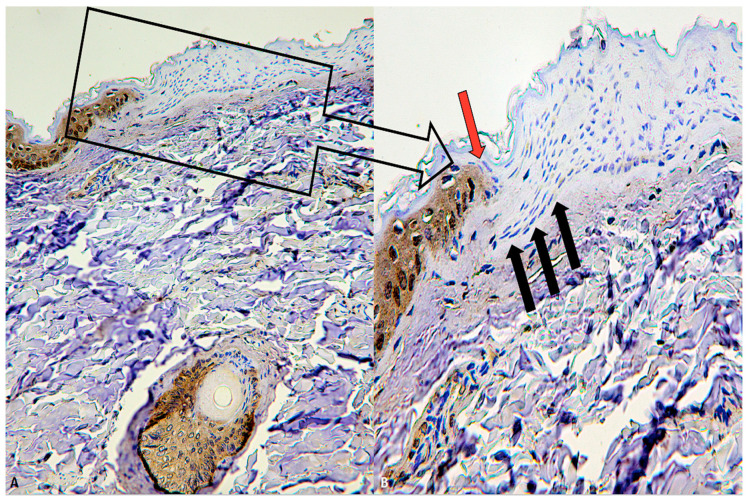
(**A**) Evident intracytoplasmic depletion of FLIP was appreciated in the epidermal layers with coexistence of epidermal flattening especially marked in the basal and spinous strati (×60). (**B**) (Insert of A) At higher magnification, the passage from the uninjured skin to the compression zone (ischemia) is clearly distinguishable (red arrow indicates the passage of the clear “ax blow” hypo-expression). Coexistence of epidermal flattening is especially marked in the basal and spinous strati (black arrows, ×200).

In all cases (21 out of 21) of subjects who died by hanging, a clear, precise and evident intracytoplasmic depletion of FLIP was appreciated in the epidermal layers with coexistence of epidermal flattening (average value of intensity −2.71, statistically significant (*p* < 0.05)) (Figure 3A,B).

**Figure 4:**
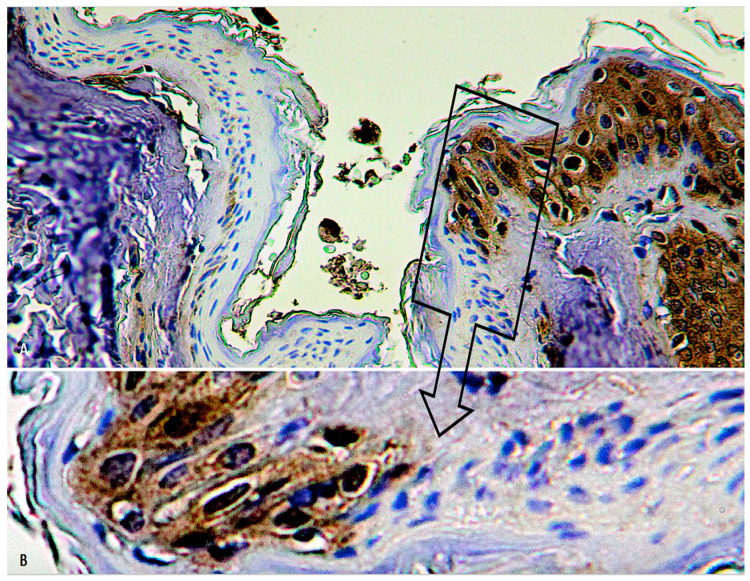
(**A**) No substantial differences in the intracytoplasmic depletion of FLIP in the epidermal layers in relation to the type of hanging (incomplete in this case) or material used (bed sheet) (×300). (**B**) Insert of (**A**) at higher magnification (×500).

We did not find substantial differences in relation to the type of hanging (complete or incomplete) or to the position of the knot or material used (Figure 4A,B) (Table 1).

**Figure 5:**
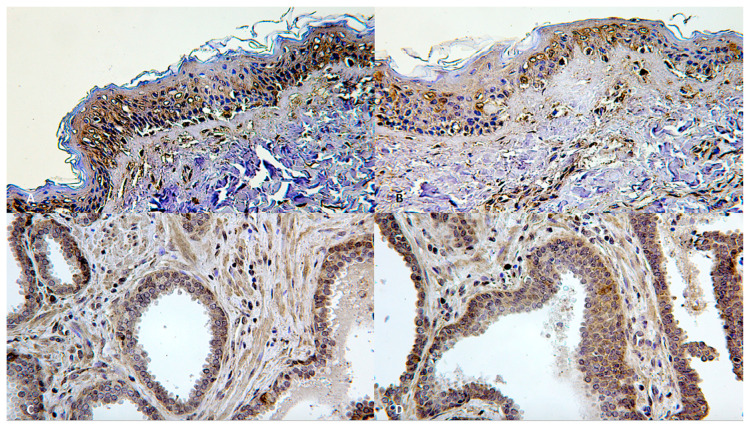
(**A**,**B**) Positive control: skin samples with normal intracytoplasmic representation of anti-FLIP (×60). (**C**,**D**) Increase anti-FLIP intracytoplasmic representation in resistant prostate cancer (×60 and ×200, respectively).

In post-mortem injuries and in uninjured skin specimens of control skin, immunohistochemical positivity was found which could not be confused with the reactions (hypo-expression) observed in vital ligature marks. The positive control showed a lack of depletion of the anti-FLIP antibody in samples of skin (Figure 5A,B) and a quantitative increase in the samples of resistant prostate cancer (Figure 5C,D).

Statistical analysis of the results via the Student test showed a statistically significant FLIP depletion for hanging compared to post-mortem injuries and in uninjured skin specimens of control skin (*p* < 0.05), and to samples of resistant prostate cancer (*p* < 0.01).

## 4. Discussion

The c-FLIP acts as an inhibitor of apoptosis induced by a variety of agents, such as tumor necrosis factor (TNF), T cell receptor (TCR), TNF-related apoptosis inducing ligand (TRAIL), and Fas and death receptor (DR) [20,21,22]. The intracellular expression of both c-FLIPL and c-FLIPS can be controlled in cancer cells at different levels. Increased expression of c-FLIP has been found in many human malignancies, such as colon, ovarian, breast and prostate cancer, as well as glioblastoma, and has been shown to be involved in resistance to CD95/Fas and TRAIL receptor-induced apoptosis [23,24,25,26].

The results of the present study are notable; in 21 cases of subjects who died by hanging, an intracytoplasmic depletion of FLIP was observed. This result confirms the intracytoplasmic reduction of c-FLIP in cervical skin at the “full of the loop” level (greater compression of the ligature mark) in subjects who died by hanging.

As shown by Dettmeyer, skin compression due to the asphyxiation medium determines a hypervascularisation of the muscular and especially of the skin structures, with consequent ischemic necrosis of the epidermis [27]. The ischemic insult would result in an under-expression of c-FLIP with activation of necroptosis and the proteolytic cascade that leads to cell death [28,29,30].

Regardless of the evidence observed during autopsy, the definitive forensic diagnosis of hanging is solely based on the finding of the vital reaction in the cervical skin sulcus. The major difficulties of interpretation are because the time of lesion formation can be extremely limited and, therefore, insufficient for the development of a clear vital reaction.

Hanging is generally performed as a means of suicide because it is an extremely simple method to implement; however, cases of suspension of a corpse are not uncommon in forensic practice. A definitive differential diagnosis between suicidal hanging and simulated hanging (hanging the victim after murder) is very delicate and critical and a challenging issue for forensic pathologists [31,32].

The skin histology is generally characterized by slight integumentary hollowing, the epidermis frequently detached from the stratum corneum, thinned, with poorly distinguishable elements, with a mainly tangential course of the cellular assises, and flattening of the interpapillary digitations [33].

Skin analyses benefit from histochemical methods, especially when using Poley staining as modified by Wenyou [34].

The immunohistochemical method is important for a correct diagnosis. The anti-fibrinogen antibodies, anti-CD15, and anti-selectin p were shown to be more reliable [16]. The anti-tryptase antibody shows a relevant positivity in cases with longer survival such as in strangulation. [17,35].

At the muscle level, the anti-myoglobin and anti-troponin antibodies are reliable indices of vitality [36]. Similar findings were reported by Fineschi et al. on the wrist, ankle, and cervical furrows caused by “incaprettamento” (18 cases): the immunohistochemical reaction (100% positivity at the neck level) was particularly effective when using anti-actin, anti-desmin, and anti-miosin polyclonal antibodies and, specifically, anti-myoglobin for muscular structures [37].

In previous studies, the possibility of a viable reaction using C5b-9, MRP14, IL-1β, and AQP3 antibodies was investigated [38,39,40].

## 5. Conclusions

Based on the results of the present study, the FLIP antibody is a trusted candidate for a definitive differential diagnosis between ante-mortem and post-mortem hangings and could be a foundation for future studies to discriminate between suicidal or simulated hanging (hanging the victim after murder). Therefore, to obtain statistically more significant and reliable results, studies with a larger number of cases are necessary.

In conclusion, the set of results obtained leads us to believe that the use of this antibody (FLIP) is very promising in being able to make a certain differential diagnosis between ante-mortem and postmortem indices of vitality.

## Figures and Tables

**Table 1:** Quantitative analysis and circumstantial data: staining intensity was evaluated using a semi- quantitative immunohistochemical scoring scale (average value of intensity −2.71, statistically significant (*p* < 0.05)). In all cases we investigated the skin at the level of the “full of the loop” (greater compression of the ligature mark).

Case Number	Gender	Staining Intensity Microscopic Results	Type of Ligature Mark	Type of Hanging	Position of Knots
1	Man	3	Soft	Complete	Occipital
2	Woman	2	Soft	Incomplete (partial)	Occipital
3	Man	3	Soft	Complete	Right/Left side of the neck
4	Man	3	Hard	Incomplete (partial)	Right/Left side of the neck
5	Man	2	Hard	Complete	Occipital
6	Man	2	Soft	Complete	Occipital
7	Woman	3	Hard	Incomplete (partial)	Occipital
8	Woman	3	Soft	Complete	Right/Left side of the neck
9	Woman	3	Soft	Complete	Right/Left side of the neck
10	Man	3	Soft	Complete	Right/Left side of the neck
11	Man	3	Soft	Complete	Right/Left side of the neck
12	Woman	2	Soft	Incomplete (partial)	Right/Left side of the neck
13	Man	3	Hard	Complete	Right/Left side of the neck
14	Man	3	Hard	Complete	Right/Left side of the neck
15	Man	2	Hard	Incomplete (partial)	Right/Left side of the neck
16	Woman	3	Soft	Complete	Occipital
17	Man	3	Hard	Complete	Occipital
18	Man	3	Hard	Incomplete (partial)	Right/Left side of the neck
19	Man	2	Soft	Complete	Right/Left side of the neck
20	Woman	3	Hard	Complete	Right/Left side of the neck
21	Woman	3	Hard	Complete	Right/Left side of the neck
